# Subacute Ruminal Acidosis as a Potential Factor that Induces Endometrium Injury in Sheep

**DOI:** 10.3390/ijms24021192

**Published:** 2023-01-07

**Authors:** Jianlin Zeng, Jianshu Lv, Hongwei Duan, Shuai Yang, Jianxin Wu, Zhenxing Yan, Rong Zhang, Junjie Hu, Yong Zhang

**Affiliations:** 1 College of Veterinary Medicine, Gansu Agricultural University, Lanzhou 730070, China; 2 Gansu Key Laboratory of Animal Generational Physiology and Reproductive Regulation, Lanzhou 730070, China

**Keywords:** claudin-1, endometrium, sheep, subacute ruminal acidosis, occludin

## Abstract

The demand for economic benefits has led to an increase in the proportion of high-concentrate (HC) feed in the ruminant diet, resulting in an increased incidence of subacute ruminal acidosis (SARA). During SARA, a high concentration of lipopolysaccharide (LPS) translocated in the rumen induces a systemic inflammatory response. Inflammatory diseases, such as endometritis and mastitis, are often associated with SARA; however, in sheep, the mechanism of the effect of SARA on the endometrium has rarely been reported. Therefore, the aim of this study was to investigate, for the first time, the influence of LPS translocation on endometrial tight junctions (TJs) during SARA in sheep. The results showed that LPS and TNFα levels in the ruminal fluid, serum, and endometrial tissue supernatant during SARA increased, transcription levels of *TLR4*, *NFκB*, and *TNFα* in the endometrium increased, the protein expression level of claudin-1 in the endometrium increased, and the protein expression level of occludin decreased. 17β-estradiol (E_2_) inhibits claudin-1 protein expression and promotes occludin expression, and progesterone (P_4_) promotes claudin-1 protein expression and inhibits occludin protein expression. E_2_ and P_4_ regulate claudin-1 and occludin protein expression through their receptor pathways. Here, we found that LPS hindered the regulatory effect of E_2_ and P_4_ on endometrial TJs by inhibiting their receptor expression. The results of this study indicate that HC feeding can cause SARA-induced LPS translocation in sheep, increase susceptibility to systemic inflammation, induce the endometrial inflammatory response, and cause endometrial epithelial TJ damage directly and/or by obstructing E_2_ and P_4_ function. LPS translocation caused by SARA has also been suggested to induce an endometrial inflammatory response, resulting in endometrial epithelial barrier damage and physiological dysfunction, which seriously affects ruminant production. Therefore, this study provides new evidence that SARA is a potential factor that induces systemic inflammation in ruminants. It provides theoretical support for research on the prevention of endometritis in ruminants.

## 1. Introduction

The epithelial layer is an initial barrier composed of epithelial cells that covers the inner and outer surfaces of various tissues, regulates substance transport and ion-selective penetration, and prevents the invasion of toxic substances [1]. This barrier function is unique to the epithelial system and is composed of tight junctions (TJs) and adherens junctions between cells. It plays a role in maintaining epithelial microenvironment homeostasis and affects cell proliferation [2]. TJ proteins are the main components of the paracellular selective barrier [3,4,5]. The 600 *k*Da tracer barrier cannot be formed in claudin-1 knockout neonatal mice [6], and the epithelial barrier function is lost in *Xenopus* embryos lacking occludin expression [7]. As pathogenic microorganisms invade and stimulate organisms, bacterial components and toxins pass through the TJ-based epithelial barrier and induce an epithelial inflammatory response and cell proliferation [1].

With the demand for economic benefits, ruminants consume an increased proportion of carbohydrates, and lower proportions of fiber in the feed decrease saliva secretion. Therefore, the small amount of saliva in the rumen is not sufficient to neutralize the significant accumulation of volatile fatty acids and lactic acid, causing a decrease in pH in the rumen and subacute ruminal acidosis (SARA) [8]. SARA, accompanied by an increase in lipopolysaccharide (LPS) concentration in the rumen, triggers an inflammatory response in the rumen epithelium and destroys the rumen epithelium barrier, which in turn causes LPS to spread to the peripheral circulation, resulting in a systemic inflammatory reaction [9]. SARA induced by a high proportion of grains causes an increase in the blood-milk barrier permeability of cows. The increase in LPS concentration in the rumen fluid, mammary artery, and mammary vein activates the NF-κB pathway in the mammary glands [10,11]. Another study found that SARA causes a decrease in endometrial immunity and an increase in TLR4 expression, and that the NF-κB pathway is activated in the endometrium [12,13]. It was suggested that LPS translocation induced by SARA could increase the inflammation susceptibility of the mammary glands and uterus and cause inflammatory reactions. However, the relevant mechanisms involved require further investigation.

Endometrial TJs play an essential role in maintaining epithelial homeostasis. Destruction of TJs results in lower uterine immunity, pathogenic microorganism susceptibility, endometrial inflammation, and an increased risk of endometrial cancer [14] and affects uterine receptivity, thus reducing sheep production performance and economic value. Progesterone (P_4_) and 17β-estradiol (E_2_) are essential sex hormones that regulate intrauterine homeostasis and cyclical uterine changes [15,16,17,18]. The transport of horseradish peroxidase (HRP) within the uterine cavity is mediated by transepithelial endocytotic activity, paracellular permeability, and the intercellular space between TJs. When injected into the uterine cavity, HRP accumulates in the cellular space under the basement membrane. This phenomenon is especially evident when the concentration of P_4_ in the circulating blood is high and the placenta of pregnant animals is attached [19,20]. A study by Min et al., showed that activation of the NF-κB pathway induced endothelial-mesenchymal transformation in patients with polycystic ovary syndrome and that E_2_ inhibited claudin-1 expression in the endometrial epithelium [21]. E_2_ and P_4_ have been suggested to regulate the intrauterine microenvironment by affecting endometrial epithelial TJ proteins.

In sheep, the mechanism by which LPS translocates to the peripheral circulation in the endometrium during SARA remains unclear. Further, whether LPS is involved in regulation of TJ proteins in the endometrial epithelium by E_2_ and P_4_ has not been reported. In order to address these gaps in the literature concerning the effects of LPS transport to peripheral circulation on sheep endometrial epithelial TJs during SARA, we established a sheep SARA model, induced inflammation from LPS in sheep endometrial epithelial cells cultured in vitro, and investigated the mechanisms of effect of sheep SARA on endometrial TJs to provide new theoretical support for research on ruminant injury.

## 2. Results

### 2.1. High-Concentrate Feed Caused SARA, and Increased LPS and TNFα Concentrations in Rumen Fluid, Serum, and Endometrial Tissue Supernatant in Sheep

**Figure 1:**
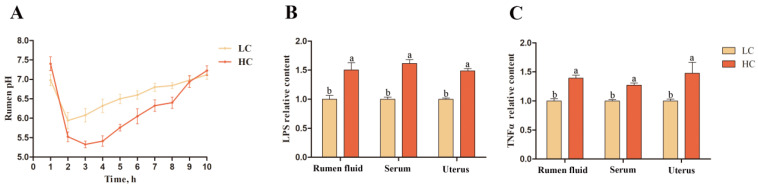
pH validation of the SARA model in sheep induced by high-concentrate feeding and comparison of the relative concentrations of LPS and TNFα in the rumen fluid, serum, and endometrial tissue supernatant of sheep in the HC group to those in the LC group. (**A**) Rumen fluid pH trend. (**B**) Relative LPS concentration. (**C**) Relative TNFα concentration. Different lowercase letters indicate a significant difference at *p* < 0.05. LC, low-concentrate; HC, high-concentrate.

The relative changes of LPS and TNFα concentrations in rumen fluid, serum, and endometrial tissue supernatant during high-concentrate-feed-induced SARA in sheep were explored. The pH trend in rumen fluid is shown in Figure 1A. Compared with the average pH of the low-concentrate (LC) group (6.6 ± 0.29), that of the high-concentrate (HC) group rumen fluid (6.2 ± 0.09) was significantly lower. In the HC group, the rumen fluid pH value remained in the 5.2–5.8 range for more than 3 h. In addition, the LPS and TNFα concentrations in the rumen fluid, serum, and endometrial tissue supernatant of the HC group were significantly higher than those in the LC group (*p* < 0.05; Figure 1B,C).

### 2.2. SARA Can Cause the Inflammatory Response of the Endometrium in Sheep 

**Figure 2:**
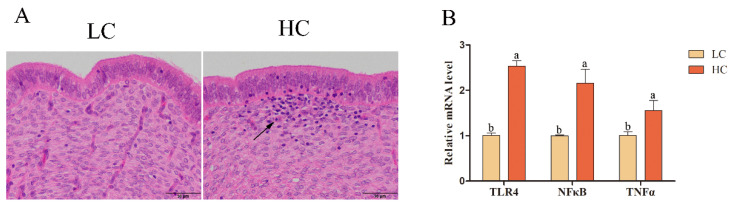
Hematoxylin-eosin staining of sheep uterine tissue sections in the LC and HC groups and relative mRNA expression levels of *TLR4*, *NFκB*, and *TNFα*. The arrows show inflammatory cells. (**A**) Hematoxylin-eosin staining. Original magnification, 400×. (**B**) Relative mRNA expression levels of *TLR4*, *NFκB*, and *TNFα*. Different lowercase letters indicate a significant difference at *p* < 0.05.

Through hematoxylin-eosin staining and qRT-PCR, we investigated whether the increase of LPS concentration in the endometrium of sheep during SARA would induce the inflammatory response. Hematoxylin-eosin staining revealed a large number of inflammatory cells in the endometrium of the HC group (Figure 2A). Further, the transcription levels of *TLR4*, *NFκB*, and *TNFα* in the endometrial tissue of the HC group increased (*p* < 0.05; Figure 2B).

### 2.3. Effects of SARA on the Endometrial Claudin-1 and Occludin Protein Expression 

**Figure 3:**
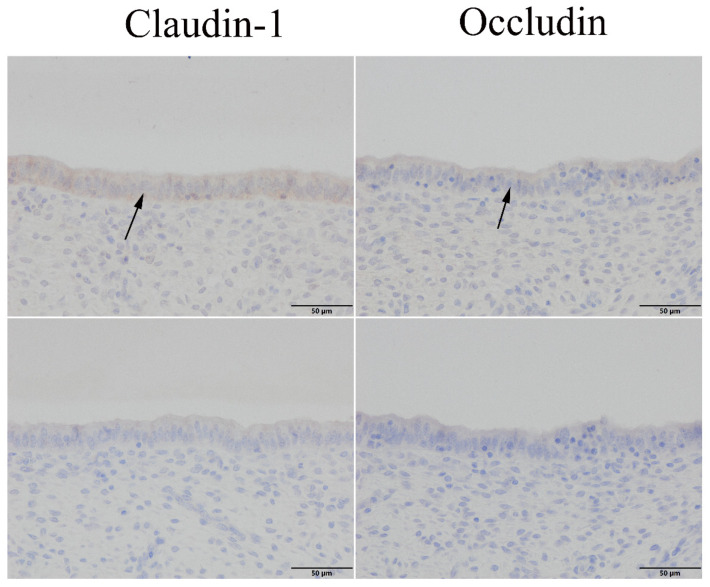
Immunohistochemical staining of claudin-1 and occludin in sheep uterine tissue samples. The arrows show the endometrial epithelium. Original magnification 400×. The superstratum is positive for claudin-1 and occludin, while the lower layer is the negative control of the same part.

**Figure 4:**
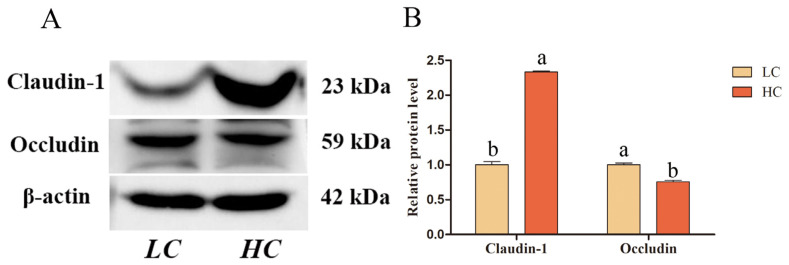
Relative protein expression levels of claudin-1 and occludin in the endometrial tissue of the HC and LC groups. (**A**) Western blotting. (**B**) Relative protein expression levels. Different lowercase letters indicate a significant difference at *p* < 0.05.

We investigated the expression sites of claudin-1 and occludin in the uterus and the effect of SARA on the expression of claudin-1 and occludin proteins in the endometrial epithelium. Immunohistochemical staining showed that claudin-1 and occludin were positively expressed in the endometrial epithelium (Figure 3). In the HC group, the relative protein expression level of claudin-1 in the endometrial epithelial tissue of the HC group increased while that of occludin decreased (*p* < 0.05; Figure 4A,B).

### 2.4. Confirmation of Primary Sheep Endometrial Epithelial Cells Cultured In Vitro 

**Figure 5:**
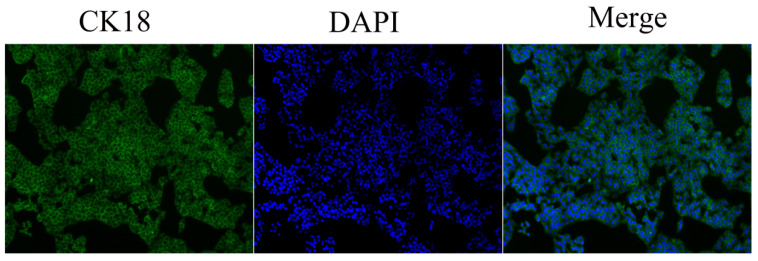
Immunofluorescence staining of cytokeratin 18 and the authentication of primary endometrial epithelial cells cultured in vitro. CK18 is fluorescent staining for cytokeratin 18. DAPI is fluorescent staining of the nucleus. Merge is CK18 staining and nucleus staining. Original magnification, 100×.

The primary endometrial epithelial cells cultured in vitro were confirmed via cellular immunofluorescence staining, and cytokeratin 18 was found to be positively expressed in the cytoplasm of the cells (Figure 5).

### 2.5. Effects of LPS-Induced Endometrial Epithelial Cell Inflammation on Claudin-1 and Occludin

**Figure 6:**
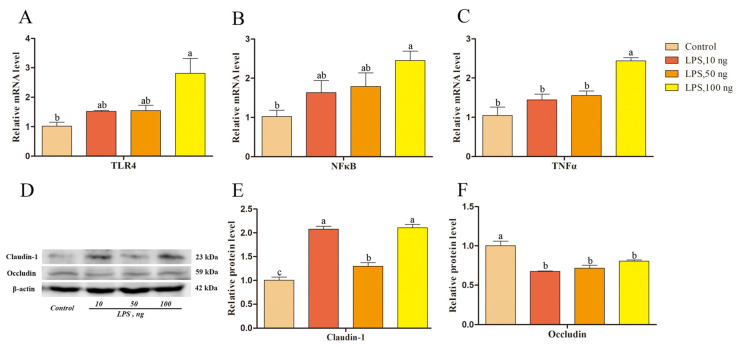
Authentication of LPS-induced inflammatory response in endometrial epithelial cells and the effect of LPS on claudin-1 and occludin protein expression in epithelial cells. (**A**) Relative *TLR4* mRNA expression level. (**B**) Relative *NFκB* mRNA expression level. (**C**) Relative *TNFα* mRNA expression level. (**D**) Western blot of claudin-1 and occludin. (**E**) Relative claudin-1 protein expression level. (**F**) Relative occludin protein expression level. Different lowercase letters indicate a significant difference at *p* < 0.05. LPS, lipopolysaccharide. Concentration is measured in ng/mL.

To explore the effect of elevated LPS in the endometrial epithelium on epithelial cells during SARA, LPS at final concentrations of 0, 10, 50, and 100 ng/mL induced endometrial epithelial cells, and *TLR4*, *NFκB*, and *TNFα* mRNA relative expression levels were determined using qRT-PCR. The results showed that LPS at a concentration of 100 ng/mL increased the transcription levels of *TLR4*, *NFκB,* and *TNFα* (Figure 6A–C). In addition, LPS concentrations of 0, 10, 50, and 100 ng/mL resulted in increased claudin-1 relative protein expression and decreased occludin protein expression (*p* < 0.05; Figure 6D–F).

### 2.6. Effects of 17β-Estradiol and Progesterone on Epithelial Cell Relative Protein Expression Levels of Claudin-1 and Occludin

**Figure 7:**
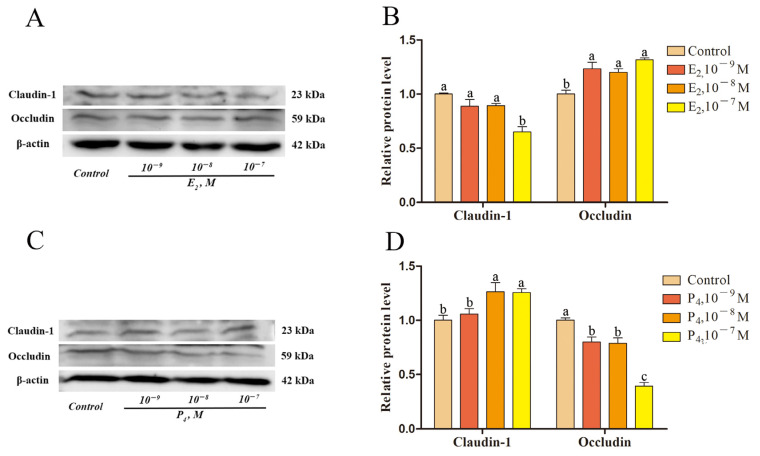
Effects of E_2_ and P_4_ on the relative protein expression levels of claudin-1 and occludin in epithelial cells. (**A**) Western blotting of E_2_ treatment. (**B**) Relative protein expression for E_2_ treatment. (**C**) Western blotting of P_4_ treatment. (**D**) Relative protein expression for P_4_ treatment. Different lowercase letters indicate a significant difference at *p* < 0.05. E_2_, 17β-estradiol; P_4_ progesterone. Concentration is measured in mol/L.

In order to explore the regulatory effects of E_2_ and P_4_ on sheep endometrial epithelial claudin-1 and occludin, epithelial cells were treated with E_2_ and P_4_ at final concentrations of 0, 10^−9^, 10^−8^, and 10^−7^ M. We found that E_2_ at a concentration of 10^−7^ M inhibited claudin-1 protein expression, while concentrations of 10^−9^, 10^−8^, and 10^−7^ M promoted occludin protein expression (*p* < 0.05; Figure 7A,B). Meanwhile, P_4_ concentrations of 10^−8^ and 10^−7^ M promoted claudin-1 protein expression and those of 10^−9^, 10^−8^, and 10^−7^ M inhibited occludin protein expression at (*p* < 0.05; Figure 7C,D).

### 2.7. Effect of SARA on the Expression of Endometrial E_2_ and P_4_ Receptors 

**Figure 8:**
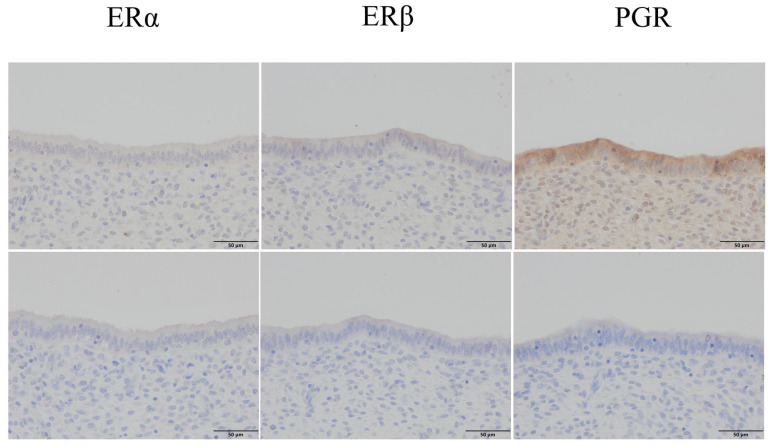
Immunohistochemical staining of ERα, ERβ, and PGR in sheep uterine samples. Original magnification, 400×. The upper layer is positive for ERα, ERβ, and PGR, while the lower layer is the negative control of the same part.

**Figure 9:**
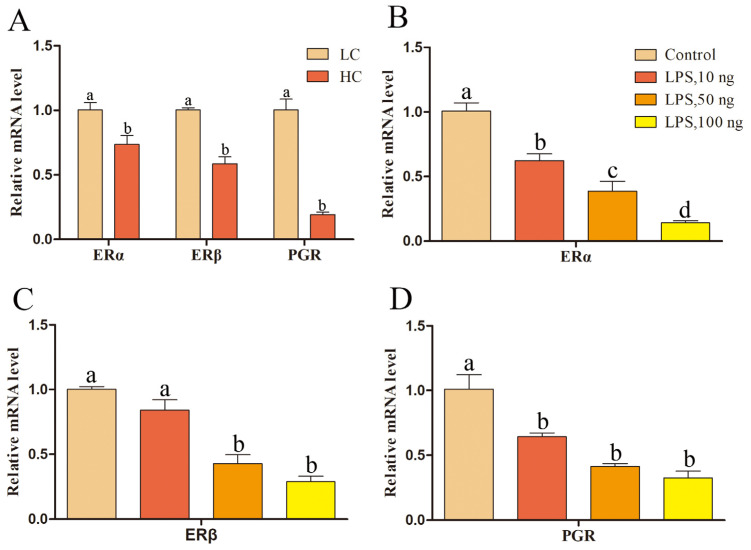
Relative *ERα*, *Erβ*, and *PGR* mRNA expression levels in endometrial tissues of the HC and LC groups and effects of LPS-induced epithelial cell inflammation on *ERα*, *ERβ*, and *PGR* mRNA expression levels. (**A**) *ERα*, *Erβ*, and *PGR* mRNA expression levels of the HC group relative to those of the LC group. (**B**) *ERα* mRNA expression level in epithelial cells. (**C**) *ERβ* mRNA expression level in epithelial cells. (**D**) *PGR* mRNA expression level in epithelial cells. Different lowercase letters indicate a significant difference at *p* < 0.05. LPS, lipopolysaccharide. Concentration is measured in ng/mL.

We explored the differences in the expression of ERα, ERβ, and PGR in sheep uterine samples during the luteal phase and the effect of LPS on the transcription levels of *ERα*, *ERβ*, and *PGR* in sheep endometrial epithelium during SARA. ERα, ERβ, and PGR were positively expressed in the endometrial epithelium (*p* < 0.05; Figure 8). The transcription levels of the E_2_ receptors *ERα* and *ERβ* and the P_4_ receptor *PGR* in the endometrial epithelial tissue of the HC group were significantly lower than those of the LC group (*p* < 0.05; Figure 9A). LPS at final concentrations of 0, 10, 50, and 100 ng/mL induced endometrial epithelial cells. Furthermore, concentrations of 10, 50, and 100 ng/mL reduced *ERα* and *PGR* mRNA expression levels (*p* < 0.05; Figure 9B,D), while those of 50 and 100 ng/mL inhibited *ERβ* transcription (*p* < 0.05; Figure 9C).

### 2.8. Mechanism of Effect of SARA on Sheep Endometrial TJs

**Figure 10:**
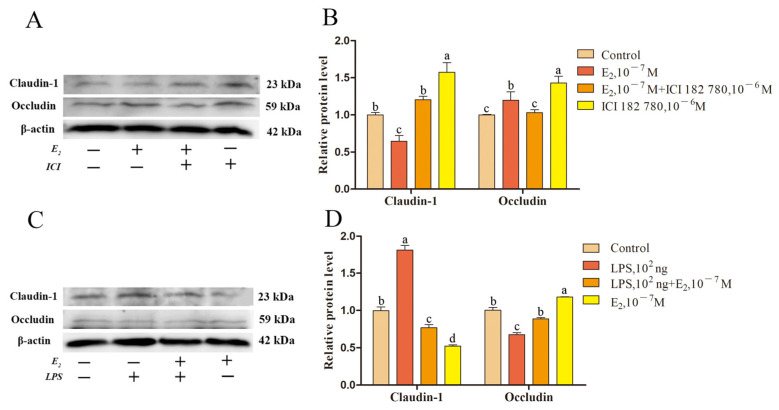
LPS affects claudin-1 and occludin protein expression in endometrial epithelial cells via E_2_ receptors. (**A**) Western blotting of E_2_ and ICI 182,780-treated epithelial cells. (**B**) Relative expression level of epithelial cell proteins treated with E_2_ and ICI 182,780. (**C**) Western blotting of LPS and E_2_-treated epithelial cells. (**D**) Relative expression level of epithelial cell proteins treated with LPS and E_2_. Different lowercase letters indicate a significant difference at *p* < 0.05. ICI 182,780, 17β-estradiol receptor inhibitor. Concentration is measured in mol/L.

**Figure 11:**
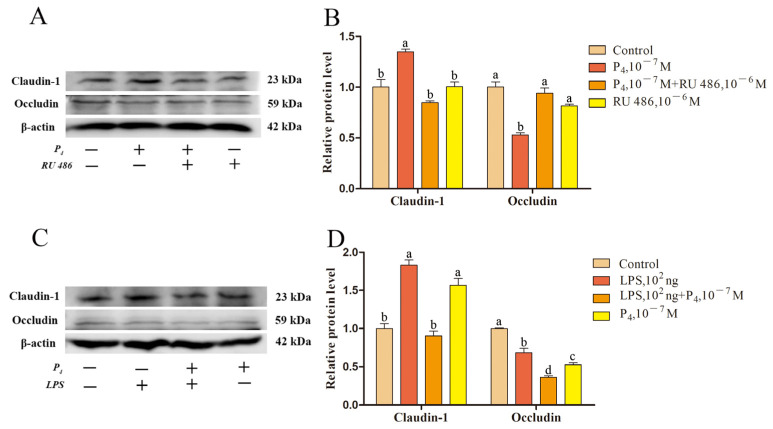
LPS affects claudin-1 and occludin protein expression in endometrial epithelial cells via P_4_ receptors. (**A**) Western blotting of P_4_ and RU 486-treated epithelial cells. (**B**) Relative expression level of epithelial cell proteins treated with P_4_ and RU 486. (**C**) Western blotting of LPS and P_4_-treated epithelial cells. (**D**) Relative expression level of epithelial cell proteins treated with LPS and P_4_. Different lowercase letters indicate a significant difference at *p* < 0.05. RU 486, progesterone receptor inhibitor. Concentration is measured in mol/L.

We investigated the effects of E_2_ and P_4_ on endometrial epithelial claudin-1 and occludin, and LPS on their functions. Treatment with ICI 182,780 at a concentration of 10^−6^ M mitigated the effect of 10^−7^ M E_2_ on claudin-1 and occludin (*p* < 0.05; Figure 10A,B). Similarly, 100 ng/mL LPS hindered the effect of 10^−7^ M E_2_ on claudin-1 and occludin proteins (*p* < 0.05; Figure 10C,D). Meanwhile, treatment with RU 486, a 10^−6^ M P_4_ receptor inhibitor, mitigated the effects of P4 on claudin-1 and occludin (*p* < 0.05; Figure 11A,B). LPS at a concentration of 100 ng/mL also inhibited the impact of 10^−7^ M P_4_ on claudin-1 and occludin proteins (Figure 11C,D).

## 3. Discussion

In this study, a sheep SARA model was established by feeding sheep a diet with a high proportion of concentrate (corn flour) and maintaining ruminal fluid pH in the range of 5.2 to 5.8 for more than 3 h a day, which is a criterion for identifying SARA [22,23], indicating that the experimental model was valid (Figure 1). Large amounts of LPS produced during SARA can disrupt rumen barrier function by inducing nitric oxide synthase expression, damaging rumen epithelial integrity, and causing the outward spread of pathogenic molecules, such as LPS, through the rumen epithelium [24,25]. There is strong evidence that LPS translocation to the peripheral circulation exacerbates SARA and increases susceptibility to systemic inflammation, causing systemic chronic inflammation [26,27,28]. In this study, LPS and TNFα concentrations in the rumen fluid, serum, and endometrial tissue supernatants of sheep in the HC group were elevated (Figure 1), leading to endometrial inflammatory cell infiltration. The transcription levels of *TLR4*, *NFκB,* and *TNFα* in endometrial tissue were increased (Figure 2). These results indicate that SARA in sheep leads to the translocation of large amounts of LPS in the rumen to the peripheral circulation, which then can stimulate the expression of TLR4 in the endometrium, activate the NFκB pathway, increase the expression of TNFα, and induce endometrial inflammation. These results confirm previous suggestions and provide new evidence of an increased risk of systemic inflammatory injury during SARA.

Claudin-1 and occludin were also expressed in the endometrial epithelium (Figure 3). They are vital transmembrane proteins that constitute epithelial TJs, maintain endometrial epithelial microenvironment homeostasis, and prevent the invasion of pathogenic microorganisms [2,3,4,5]. Compared to that in the LC group, the protein expression level of claudin-1 in the endometrial tissue of the HC group was higher while that of occludin was lower (Figure 4). Similar results were obtained in the LPS-induced endometrial epithelial cell inflammation model (Figure 6). These results indicate that LPS translocated to the peripheral circulation during SARA can stimulate the inflammatory response of the sheep endometrium, thereby causing an increase in the expression of claudin-1 protein and a decrease in the expression of occludin in the TJ of the epithelium. It is suggested that the endometrial inflammation caused by LPS in the peripheral circulation during SARA in sheep will cause endometrial epithelial TJ damage and increase endometrial susceptibility. TNFα is a multi-effect inflammatory factor produced by macrophages and monocytes that can cause an inflammatory cascade by triggering the expression of other proinflammatory factors, with excessive activation of endometrial epithelial TJ neutrophils causing epithelial damage [29,30,31]. Previous studies have reported that TNFα-induced Alport mice and horses showed increased claudin-1 expression in renal tubular TJs [32,33], while decreased occludin expression was found in LPS-induced canine endometritis [34]. Endometrial occludin protein and mRNA expression levels were found to be significantly reduced in *E. coli*-treated mice [35]. These reports directly indicate that inflammation and barrier function are closely associated.

E_2_ causes endometrial epithelial and stromal proliferation in ewes and baboons undergoing ovariectomy and uterine glands and microvessels to increase in volume [36,37,38,39,40]. Claudin-1 knockout causes skin dehydration [6,41], blood-brain barrier [42], myelin sheath and a supporting cell layer [43], and epithelial cells in the inner ear tightening [44]. Many studies have shown that the amount of occludin expressed in tissues is inversely proportional to its paracellular permeability [16,45,46,47,48]. Proestrus and estrus rats with estrogen predominance have dilated uterine cavities and contain large amounts of fluid, are progestogen-predominant post-estrus, and have diestrus uterine cavity reduction [16,18]. This study showed that E_2_ inhibited claudin-1 expression and promoted occludin expression while P_4_ had the opposite effect (Figure 7). E_2_ and P_4_ played different roles in the regulation of claudin-1 and occludin in the sheep endometrial epithelium, just as the uterus showed different states in different sex-hormone-dominant periods in the above study. Changes in the ratio of E_2_ and P_4_ in different estrous cycles may be one of the reasons for different states of the intrauterine environment. This suggests that E_2_ and P_4_ affect the expression of endometrial epithelial TJ proteins, regulate endometrial epithelial cell proliferation and bypass permeability, and participate in the physiological regulation of the sheep uterus.

We analyzed the transcription levels of E_2_ receptors *Erα* and *ERβ* and the P_4_ receptor *PGR* in the endometrial epithelium of sheep (Figure 8) and found that the levels decreased due to SARA-induced inflammation of the endometrial epithelium. Similar results were obtained for an LPS-induced model of epithelial cell inflammation (Figure 9). During SARA, LPS can inhibit *ERα*, *ERβ*, and *PGR* expression in the endometrial epithelium, affecting the regulatory functions of E_2_ and P_4_ in the endometrial epithelium. Studies on human vascular endothelial cells have found that E_2_ can increase occludin expression levels through its receptors and can reduce paracellular permeability [45]. Ovariectomy rat E_2_ lowers colon cell permeability in a dose-dependent manner, and this effect is inhibited by ICI 182,780 [18]. Meanwhile, P_4_ treatment reduces the occludin protein expression in the sheep endometrium, and cotreatment with RU 486 increases occludin protein expression [49].

In this study, we found that E_2_ and P_4_ regulate the expression of the endometrial epithelial TJ proteins claudin-1 and occludin through their receptor pathways. LPS stimulated the epithelium, which caused a decrease in the expression of *ERα*, *ERβ*, and *PGR*, resulting in the regulation of claudin-1 and occludin by E_2_ and P_4_ (Figure 10 and Figure 11). LPS has been shown to stimulate the inflammatory response of endometrial epithelial cells, directly destroy epithelial TJs, or inhibit E_2_ and P_4_ receptor expression, affecting E_2_ and P_4_ function which ultimately leads to damage of the endometrial epithelial microenvironment. It is suggested that LPS in the peripheral circulation during SARA in sheep can cause damage to endometrial TJs through the middle pathway, resulting in decreased endometrial function and increased susceptibility risk in sheep.

In conclusion, this study showed that SARA induced by a high concentration grain diet in sheep led to increased LPS concentration in rumen fluid, translocation of LPS to peripheral circulation, and increased susceptibility to systemic inflammation. LPS translocation can induce endometrial epithelial inflammation directly and/or by inhibiting *ERα*, *ERβ*, and *PGR* expression to cause E_2_ and P_4_ dysfunction, damaging epithelial TJs. It was suggested that an increase in the concentration of peripheral circulating LPS during SARA in ruminants caused an endometrial inflammatory response. The expression of endometrial epithelial claudin-1 and occludin is affected by different pathways, which increases the permeability of the epithelium, impairing the epithelial barrier function and endangering the safety of the intrauterine environment. Therefore, the systemic response induced by LPS translocation during SARA requires attention, and the effects of endometrial epithelial TJ injury on uterine receptivity, embryo implantation, and embryonic development warrant further research. This study made a preliminary exploration on the influence of SARA on the reproductive system of ruminants, providing new ideas for prevention and treatment of reproductive system diseases and reducing inflammatory susceptibility, and contributing to further improving the economic output value of ruminants.

## 4. Materials and Methods

### 4.1. Establishment of a SARA Model

All experimental procedures were approved by the Animal Care and Use Committee of the College of Veterinary Medicine, Gansu Agricultural University, Lanzhou, Gansu, China. The experiment was carried out during the period of anestrus in the sheep. Sexually mature but never-mated grass-fed healthy female sheep purchased from local farmers (*n* = 16; 7 ± 1 months old; 25 ± 5 kg) were pre-fed with corn flour and alfalfa (2:8) for one week before the start of the experiment. They were randomized into two groups: the low-concentrate (LC) group (*n* = 8), wherein the ratio of corn flour to alfalfa was adjusted to 7:3, and the high-concentrate (HC) group (*n* = 8), wherein the ratio remained unchanged. Rumen fluid was extracted from the rumen at 1 h intervals for 10 h on days 5, 6, and 7 of the 10th week of the experiment. The rumen fluid was collected by a gastric tube sheep rumen fluid sampler (MDW16; Chengdu Huazhi Kaiwu Technology Co., Ltd., Chengdu, China). In order to avoid the influence of saliva on the pH of the rumen fluid, the rumen fluid collected for the first time was discarded, and the subsequent samples were filtered through four layers of gauze. The pH of the rumen fluid was measured immediately using a pH meter (Sartorius, Goettingen, Germany). Serum and rumen fluid were collected after 10 weeks, and the uteri of luteal stage sheep (LC, *n* = 5; HC, *n* = 4) were collected after killing for subsequent experiments.

### 4.2. Enzyme-Linked Immunosorbent Assay

Recently collected sheep endometrial tissue (0.5 g; LC: *n* = 5, HC: *n* = 4) was weighed and homogenized at 4 °C with 100 μL of phosphate buffer (PBS). The tissue homogenate, serum, and rumen fluid were centrifuged at 3000 rpm at 4 °C for 20 min to collect the supernatant. Absorbance was measured at 450 nm according to the manufacturer’s instructions, and a standard curve was constructed to determine the relative concentrations of LPS (YFXESh00066; Yifeixue Bio Tech, Nanjing, China) and TNFα (YFXESh00022; Yifeixue Bio Tech).

### 4.3. Hematoxylin-Eosin Staining

Uterine tissues were fixed in 4% (*v*/*v*) paraformaldehyde with 0.1 M PBS (pH 7.4) and embedded in paraffin. Sections of 4 μm thickness were mounted onto gelatin/poly L-lysine-coated glass slides and dried in an incubator at 60 °C for 2 h. Paraffin sections were submerged in hematoxylin dyeing solution (D10393; Bioss, Beijing, China) for 2 min, 1% Acid Alcohol Fast Differentiation Solution (C0163M; Beyotime, Haimen, China) for 3 s, and eosin (S0159; Bioss) for 1 min. The sections were then processed through gradient alcohol and xylene dehydration. Sections were observed and photographed using an Olympus-DP73 optical microscope (Olympus, Tokyo, Japan).

### 4.4. Immunohistochemical Staining

Uterine tissues were fixed in 4% (*v*/*v*) paraformaldehyde with 0.1 M PBS (pH 7.4) and embedded in paraffin. Sections of 4 μm thickness were mounted onto gelatin/poly L-lysine-coated glass slides and dried in an incubator at 60 °C for 2 h. They were then processed through xylene dehydration and gradient alcohol dewaxing. An immunohistochemical streptavidin-peroxidase conjugate kit was used (SP−0023 and SP−0024; Bioss) following the manufacturer’s instructions. The sections were incubated overnight at 4 °C with antibodies (diluted 1:100) against claudin-1 (28674–1-AP; Proteintech Group, Wuhan, China), occludin (66378–1-Ig; Proteintech Group), ERα (ab−66102; Abcam), ERβ (ab−3576; Abcam), and PGR (bs−23376R; Bioss), after which they were washed with PBS. Sections were incubated with streptavidin-peroxidase (sp−0023 and sp−0024; Bioss) at 37 °C for 15–20 min and then washed with PBS. Next, the sections were incubated with HRP-labeled streptavidin (sp−0023 and sp−0024; Bioss) for 15–20 min at 37 °C, and a positive signal was observed using DAB (D10294; Bioss). The paraffin sections were washed and immersed in hematoxylin dyeing solution (D10393; Bioss) for 10–20 s, after which the dye was removed with water. The sections were then processed through gradient alcohol and xylene dehydration. For the negative control, PBS was used instead of primary antibodies for incubation, following the procedure described above. Sections were observed and photographed using an Olympus-DP73 optical microscope (Olympus).

### 4.5. Total RNA Isolation and Quantitative Reverse Transcription Polymerase Chain Reaction (qRT-PCR)

Total RNA was extracted from endometrial tissue and cultured endometrial epithelial cells using TRIzol reagent (Solarbio, Beijing, China) and was reverse-transcribed using the Prime Script RT Reagent Kit with a cDNA Eraser (Takara Bio, Dalian, China). mRNA levels were determined via qRT-PCR using the LightCycler 480 Real-Time Detection System (Roche, Basel, Switzerland). The reaction mixtures consisted of 10 μL of 2 × SYBR Green II PCR Mix (Takara Bio), 25 μmol/L each of forward and reverse primers, 2 μL of template, and distilled water up to a final volume of 20 μL. The reaction conditions were as follows: 95 °C for 30 s, followed by 40 cycles of 95 °C for 10 s, 60 °C for 20 s, and 72 °C for 10 s. A melting curve was obtained from 65 °C to 95 °C, increasing at intervals of 0.5 °C every 5 s. β-actin was used as an internal control. Four replicates were used for each sample to ensure the accuracy of the relative expression of target genes. The primers used were designed based on the mRNA sequence data available in GenBank; TLR4 [50], NFκB, TNF-α, Erα [51,52], ERβ, PGR [53], β-actin [52], and the sequences are listed in Table 1. The relative expression of target genes was calculated using the 2^−ΔΔCT^ method [54].

### 4.6. Western Blotting

Cells and tissue samples were lysed using a radioimmunoprecipitation assay buffer (Solarbio) containing 1:100 (*v*/*v*) phenylmethylsulfonyl fluoride (Solarbio) and were then centrifuged at 12,000 rpm for 5 min at 4 °C to obtain protein samples. The protein concentration was determined using a Bicinchoninic Acid Protein Assay Kit (Solarbio). Protein (40 μL) was mixed with 4 × loading buffer (Solarbio) and separated using 12% sodium dodecyl sulfate-polyacrylamide gel electrophoresis. The separated proteins were then transferred onto polyvinylidene fluoride membranes (Millipore, Atlanta, GA, USA) and washed with Tris-buffered saline with Tween 20 (TBST; Solarbio) at room temperature for 30 min. The membranes were blocked with 5% skim milk in TBST at room temperature for 30 min and incubated with antibodies against claudin-1 (28674–1-AP; Proteintech Group) and occludin (66378–1-Ig; Proteintech Group) at 4 °C for 8 h, with β-actin (diluted 1:3000; bs−0061R; Bioss) as an internal reference. The membranes were then washed and incubated with HRP-conjugated AffiniPure goat anti-rabbit IgG (H + L) (diluted 1:4500; SA00001–2; Proteintech) or HRP-conjugated AffiniPure goat anti-mouse IgG (H + L) (diluted 1:4500; SA00001–1; Proteintech). Immunoreactivity was observed using enhanced chemiluminescence (Abnova, Taipei, Taiwan), and the signal was quantified using ImageJ 10.0 software (National Institute of Health, Bethesda, MD, USA).

### 4.7. Endometrium Epithelial Cell Culture and Treatment

To further investigate the effect of elevated LPS concentrations during SARA on the endometrial epithelium TJs, the sheep uterus of the LC group was collected and primary endometrial epithelial cells were cultured [55]. Antibiotics (50 IU/mL penicillin and 50 IU/mL streptomycin; Solarbio) at 37 °C in PBS and 75% alcohol were used to sequentially wash the endometrial tissue thrice, and the endometrial epithelium was cut with 0.2% collagenase type 4 (Sigma-Aldrich, St. Louis, MO, USA) at 37 °C for 10 min. Centrifugation was performed at 1200 rpm for 5 min to remove the supernatant, followed by washing with PBS thrice. The cells were then cultured in Dulbecco’s modified Eagle medium (DMEM)/F12 complete medium suspension containing 100 IU/mL penicillin (Solarbio), 100 IU/mL streptomycin (Solarbio), and 10% fetal bovine serum at 37 °C with 95% O_2_ and 5% CO_2_ in a humidified incubator. The medium was changed every 2 d, and the cells were split upon reaching 80–90% confluency and cultured in six-well plates (Corning, Glendale, AR, USA), which contained 2 mL of medium. During treatment, DMEM/F12 was added to the original culture for 12 h and was used to treat according to the test needs for 24 h, after which the supernatant was discarded, and the cells were washed with PBS precooled at 4 °C three times and stored at −80 °C until use.

### 4.8. Immunofluorescence

Cytokeratin is a component of the cytoskeleton, mainly present in all epithelial cells and some non-epithelial cells, and is used to maintain the shape of cells and resist external stress. CK18 and its type II pair CK8 are probably the most common products of the intermediate fiber gene family. For example, in some highly specialized parenchyma epithelial cells and in some endocrine cells and normal liver cells, CK8 and CK18 are the only keratin proteins present [56,57]. Therefore, primary cultured endometrial epithelial cells were identified by CK18 immunofluorescence staining. The culture medium was discarded when the cell density in the six-well plate (Corning) reached 70–80% confluency. The cells were washed with prechilled PBS at 4 °C, fixed with 4% (*v*/*v*) paraformaldehyde, washed again with PBS, and then treated with 0.1% Triton x−100 (Solarbio) at room temperature for 15 min, followed by a final wash with PBS. The cells were incubated with 5% goat serum at 37 °C for 30 min and incubated overnight at 4 °C with antibodies against cytokeratin 18 (diluted 1:300). The following day, the cells were washed with PBS and incubated with fluorescein isothiocyanate-conjugated goat anti-rabbit IgG (H + L) antibody (ab150077; Abcam) for 45 min, after which the nuclei were counterstained with 1 μg/mL 4′,6-diamidino−2-phenylindole. Images were acquired using a Revolve Omega fluorescence microscope (Apex Bio, Houston, TX, USA).

### 4.9. Statistical Analysis

Statistical analyses were performed using SPSS 21.0 (IBM Corporation, Armonk, NY, USA). Quantitative data are presented as the mean ± standard error of the mean. All data were tested for normality and homoscedasticity and subjected to one-way analysis of variance followed by Duncan’s multiple range test. Statistical significance was set at *p* < 0.05.

## Figures and Tables

**Table 1:** Forward and reverse primers.

Genes	Sequences (5′−3′)	Accession No.
TLR4	F: GGTTTCCACAAGAGCCGTAA	NM_001135930.1
	R: CTGTTCAGAAGGCGATAGA	
NFκB	F: CCAGCATCAAAATCCCCAGC	EF121765.1
	R: GTGAAGGGTTGGAGACCTCA	
TNF–α	F: GGGAACACAGACAGAGGGGACA	EF446377.1
	R: CCTGCGAGTAGATGAGGTAAAG	
ERα	F:GACCGAAGAGGAGGGAGAATG	AY033393.1
	R:CGGGCTGTTCTTCTTAGTGTGTT	
ERβ	F:ACACCTCTCTCCTTTAGCCATCC	AF177936.1
	R:TCCTTTTCAATGTCTCCCTGTTC	
PGR	F:GTCAGGCTGGCATGGTTCTT	Z66555.1
	R:GGGCTTGGCTTTCATTTGG	
β-actin	F: GTCACCAACTTGGGACGACA	U39357
	R: AGGCGTACAGGGACAGCA	

## Data Availability

The datasets produced and/or analyzed during the current study are available from the corresponding author upon reasonable request.
